# Ultrasound of living donor liver transplantation

**DOI:** 10.2349/biij.2.2.e17

**Published:** 2006-04-01

**Authors:** L Leong

**Affiliations:** Department of Radiology, Queen Mary Hospital, Hong Kong SAR, China

**Keywords:** Ultrasound, living donor, liver, transplantation

## Abstract

Liver transplantation is the most effective treatment for various end-stage liver diseases. Living donor liver transplantation (LDLT) was first developed in Asia due to the severe lack of cadaveric graft in this region. The Liver Transplant Service at Queen Mary Hospital (QMH), Hong Kong, has pioneered the application of LDLT to patients using both left lobe and right lobe grafts. The QMH liver transplant programme is the largest of its kind in China and Southeast Asia.

Ultrasound (US) is often employed in the initial work-up of potential donor and recipient of LDLT. It is the imaging technique of choice to assess the early and late complications of LDLT, with colour Doppler ultrasound being the most useful in the evaluation of post-LDLT vascular complications. The use of ultrasound contrast agents improves the visualisation of the hepatic vasculature, possibly delaying or removing the need for more invasive investigations. Intra-operative ultrasound facilitates the determination of the resection plane during donor hepactectomy.

Computed tomography (CT) or magnetic resonance imaging (MRI) can be used as the single imaging modality in the evaluation of LDLT candidates.

Ultrasound is most useful as the initial screening test in detecting hepatic parenchymal abnormalities, while CT or MRI is the modality of choice in the demonstration of vascular and biliary anatomy of the potential liver donor.

Biliary complications are more common in LDLT than in cadaveric liver transplantation. The ductal dilatation, resulting from biliary stricture, is clearly demonstrated by ultrasound. Bilomas can be aspirated under ultrasound guidance to confirm the diagnosis and to promote healing. Perihepatic fluid collections and abscesses are also common after LDLT. Intra-hepatic collections may represent seromas, haematomas or infarction. Ultrasound is a sensitive means of detecting these collections and can be employed to guide drainage in suitable patients.

Transplant-related malignancies include recurrent neoplasia and post-transplant lymphoproliferative disease (PTLD). Ultrasound can be used to screen for recurrent disease and to detect PTLD in the transplanted liver.

## INTRODUCTION

Liver transplantation is the most effective treatment for various end-stage liver diseases and sudden, acute liver failure. Due to the worldwide shortage of cadaveric liver, innovative surgical techniques, including split liver transplantation and living donor liver transplantation (LDLT), have been developed. LDLT was first initiated in Asia to address the severe lack of cadaveric graft in this region. Over 12 years, more than 1500 cases of LDLT have been performed in five Asian centres (Kyoto, Tokyo, Asan, Hong Kong, Taiwan) with 1- and 5-year actuarial patient survival rates of 78.7%-97.8% and 76.1%-97.8% being achieved, respectively [1].

The Liver Transplant Service at Queen Mary Hospital (QMH), Hong Kong, has pioneered the application of LDLT to patients using both left lobe and right lobe grafts. The QMH liver transplant programme is the largest of its kind in China and Southeast Asia. By the end of December 2003, a total of 321 liver transplants (210 right lobe LDLT) were performed with 1-year and 5-year survival rates of 84% and 79%, respectively. Successful transplantation requires a detailed evaluation of the transplant candidate and the potential living donor.

Imaging has contributed to the development and success of LDLT in the selection of patients as well as detection and management of post-transplant complications. The use of ultrasound in the evaluation of LDLT patients and assessment of the post-operative complications is discussed in this review.

## I. PRE-TRANSPLANT ASSESSMENT

### Evaluation in recipients

Pre-transplant imaging plays an important role in identifying contraindications to transplantation, anatomic abnormalities and variants that may alter the surgical approach.

#### i) Liver parenchyma

Ultrasound may show changes of cirrhosis with nodular contours, parenchymal inhomogeneity, right- lobe atrophy and hypertrophy of lateral segment and caudate lobe [2].

Cirrhosis often causes narrowing of the hepatic veins with loss of the normal phasic waveform. Intra-hepatic vessels may be indistinct. The caudate lobe can become enlarged and surround the inferior vena cava (IVC), which is of relevance in cases of living donor transplantation in which IVC is preserved.

Colour Doppler ultrasound may show portal vein flow reversal or portal collaterals, suggesting the diagnosis of portal hypertension.

#### ii) Presence and extent of hepatocellular carcinoma

Liver transplantation for the treatment of hepatocellular carcinoma (HCC) provides excellent outcomes with application of the Milan criteria (single nodule < or = 5cm, or two or three nodules < or = 3cm) with 5-year survival rates of 70% and low recurrence rates [3].

A recent study has suggested that currently available selection criteria for HCC patients can be applicable to LDLT without change of prognostic power [4].

**Figure 1:**
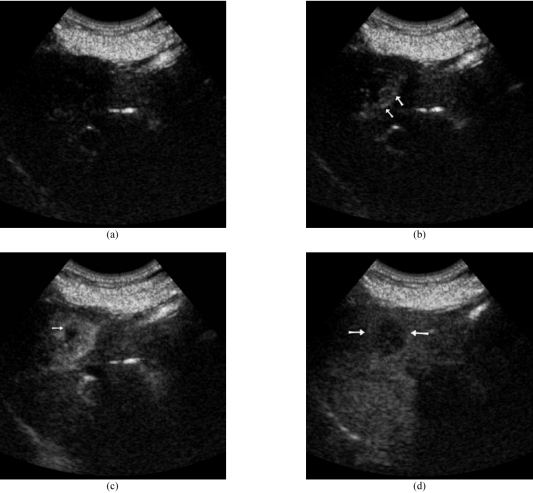
Hepatocellular carcinoma (HCC) in a 75 year old woman. (a) Baseline US showed a hypoechoeic lesion at the right hepatic lobe. (b) Dynamic contrast enhanced US with SonoVue. The early arterial phase image demonstrated peripheral tumoural vessels (arrows) with enhancement filling from the periphery. (c) The arterial phase image showed homogeneous tumoural enhancement with a small hypoechoic area (arrow). (d) In the portal phase, the HCC (arrows) became relatively hypoechoic to the surrounding enhanced liver parenchyma.

Ultrasound is often used to screen HCC in a high risk population. Contrast enhanced ultrasound greatly increases the sensitivity in the detection of HCC (Figure 1) [5].

Accurate radiological staging is required to define suitable HCC candidates for liver transplantation. While either computed tomography (CT) or magnetic resonance imaging (MRI) can be used, many studies have found MRI to be superior to detect and characterise focal hepatic lesions [6].

#### iii) Presence of cholangiocarcinoma and other malignancies

Known cholangiocarcinoma is a contraindication to liver transplantation due to a high recurrence rate. The detection of an extra-hepatic tumour with or without hepatic metastasis also excludes the patient from transplantation. Ultrasound evaluates the intrahepatic tumour. Both CT and MRI can also be used to depict extra-hepatic disease in patients with malignancy, with studies showing MRI to be superior to helical CT in this regard [7].

#### iv) Patency of the portal vein and superior mesenteric vein

Diffuse thrombosis of the portal and superior mesenteric vein (SMV) is a contraindication to liver transplantation. Portal vein thrombosis requires the modification of surgical technique at the time of transplantation. Ultrasound can be used to assess the vascular patency of a potential transplant recipient. Contrast enhanced ultrasound improves the colour and spectral Doppler signal, thereby increasing operator confidence. However, ultrasound evaluation of the SMV patency remains problematic because of overlying bowel gas. Contrast enhanced CT or MRI/MRA (magnetic resonance angiography) can be employed to evaluate the patency of these vessels.

#### v) Celiac artery stenosis

Recipients with celiac artery stenosis are at risk of compromised blood flow to the transplanted organ. Doppler ultrasound has high accuracy in the diagnosis of high grade splanchnic arterial stenosis, and can be used as a screening method for celiac artery stenosis [8].

Computed tomography angiography (CTA) is an established method to evaluate arterial anatomy, aneurysm and stenosis [9].

Narrowing, mural thrombus and post-stenotic dilatation can be demonstrated with CTA. Contrast enhanced MRA is the alternate means of assessing these abnormalities. The detected stenosis is corrected at surgery.

#### vi) Status of transjugular portosystemic shunt

Some recipients may have undergone placement of a transjugular portosystemic shunt (TIPS) prior to transplantation. The patency of the shunt can be assessed with colour Doppler ultrasound, including Power Doppler. The use of ultrasound contrast readily establishes the patency of the stent. Migration of the shunt and shunt related stenosis could affect transplantation in a significant number of patients. The location of the proximal and distal ends of the shunt can easily be determined by CT. However, MRI evaluation of graft patency may be limited by metallic stent artefact.

### Evaluation in liver donors

In addition to detailed clinical examination, informed consent with the surgical team, cardiopulmonary function assessment and numerous laboratory and serologic tests, the potential living donor has to undergo extensive imaging evaluation to identify exclusion criteria for liver donation and obtain necessary information to ensure success in transplantation.

#### i) Hepatic artery anatomy to the graft lobe

Detailed anatomic information is required to plan the hepatic-arterial, portal-venous and hepatic-venous surgical anastomosis in LDLT. Hepatic angiography is the traditional gold standard in the preoperative assessment of the vascular anatomy of potential liver transplant patients. Multi-detector computed tomography (MDCT) or contrast-enhanced MRI can be used as a non-invasive alternative (10, 11, 12).

Intra-operative ultrasound is performed to direct hepatic transection in an avascular plane during donor hepactectomy.

The disadvantages of CT include the use of ionising radiation and injection of potentially nephrotoxic iodinated contrast.

#### ii) Venous and biliary anatomy

The main branches of the hepatic vein drain into the inferior vena cava. The portal vein usually bifurcates into the left and right portal veins, which are easily demonstrated by Doppler ultrasound. The main portal vein may “trifurcate” with an early branching pattern in the right hepatic lobe. Accessory right hepatic veins occur in 6% of people and can cause increased bleeding if unrecognised before surgery.

Venous anatomy is well demonstrated by contrast enhanced CT or MRI/ MRA.

The biliary system can be evaluated with endoscopic retrograde cholangiopancreatography (ERCP), CT cholangiography [13] and MR cholangiography (MRC). MRC, utilising heavily T2W pulse sequences, accurately depicts biliary anatomy in potential LDLT donors and guides the intra-operative management of the biliary tract [14].

MRC, enhanced with hepatobiliary agent, is highly accurate in demonstrating the biliary anatomy in adult living liver donors [15].

#### iii) Liver volume

The success of LDLT depends on adequate graft volume. The volumes of potential donor livers are matched with the volume needed by the recipient, as calculated according to body surface area. A minimum of 40% of the normal liver volume is required by the recipient [16]. At least 30% of the liver should be left in the donor [17].

Both CT and MRI can be used to calculate the graft and remnant liver volume. CT provides accurate and reproducible estimate of liver graft mass. Besides hepatic volume determination, MRI can also estimate the degree of fatty infiltration and evaluate the vascular and biliary anatomy, without the risk of ionising radiation.

#### iv) Liver parenchyma

An important donor-related risk factor, associated with poor recipient outcome after orthotopic liver transplantation, is moderate- to severe donor steatosis.

Ultrasound, CT and MRI are useful to evaluate focal hepatic lesions detected in asymptomatic potential donors.

Ultrasound easily detects the presence of fatty infiltration. Severe fatty infiltration often results in an enlarged liver with diffuse increase echogenicity [2].

Acoustic penetration may be reduced with indistinctness of the blood vessels and the diaphragm. Quantification of the degree of fatty infiltration may be accomplished with dual energy CT.

Opposed-phase gradient echo MRI can be performed to provide an objective estimate of the degree of hepatic steatosis. Donor hepatic biopsy is performed in some institutions to accurately assess the degree of steatosis.

## II. INTRA-OPERATIVE EVALUATION

Intra-operative ultrasound is used to determine the optimal plane of resection during LDLT. The donor liver must be divided along a plane that avoids major vessels, while leaving both donor and recipient with sufficient liver volume for proper function. The optimal resection plane is about 1 cm to the right of the middle hepatic vein [18].

Our Centre now employs the hepatic venoplasty technique to facilitate the implantation of the right lobe graft and quarantines flow in the middle hepatic vein [19].

Intra-operative ultrasound is most useful to depict venous anatomy for the surgeon. The hepatic vein branches, from segment V and VIII, that drain into the right hepatic vein are identified by ultrasound and marked, because these vessels will be divided during donor hepatectomy [20].

Accessory hepatic veins can also be identified by ultrasound. The right portal vein should not be divided too close to the portal vein bifurcation, otherwise a stenosis will be created [21].

Ultrasound can be used to identify the origin of the right portal vein during right lobe resection. Intra-operative Doppler ultrasound also helps detect abnormal hepatic hemodynaemics, allowing early intervention to ensure better patient outcome (22, 23).

## III. NORMAL ULTRASOUND APPEARANCE OF LIVER TRANSPLANTS

A routine post-operative ultrasound of the transplanted liver includes grey-scale assessment of the liver parenchyma and biliary tree and Doppler study of the hepatic vasculature. The normal portal vein Doppler waveform is a continuous flow pattern toward the liver with mild velocity variations induced by respiration. The normal Doppler appearance of the hepatic veins and IVC shows a phasic flow pattern.

The normal graft liver has a homogeneous or slightly heterogeneous pattern at grey-scale imaging [24]. The intrahepatic biliary ducts should be of normal caliber and appearance. In the early post-operative period there is usually a small amount of perihepatic fluid. The normal hepatic artery Doppler waveform shows a rapid systolic up-stroke with continuous diastolic flow. The acceleration time should be less than 80 msec, and the resistive index (RI) should be between 0.5 and 0.7 [24].

High-resistance flow at the hepatic artery detected on Doppler ultrasound during the period immediately after transplantation is a frequent finding and has no prognostic implication [25].

## IV. EVALUATION OF POST-LDLT COMPLICATIONS

Despite the rapid advances in surgical techniques and improvements in immuno-suppression, various complications still occur after LDLT, resulting in significant patient morbidity and mortality (1,26,27) (Table 1).

**Table 1:** Complications of liver transplantation at Queen Mary Hospital, Hong Kong (October 1991-December 2003)

**Major complication**	**Percentage**	**Number of patients** **n = 321**
Delayed graft dysfunction	0.3%	1
Primary non-function	0.6%	2
Post-transplant lymphoproliferative disease	1.9%	6
Graft versus host disease	0.6%	2
Hepatic vein stenosis/ thrombosis	1.9%	6
Hepatic artery thrombosis	2.2%	7
Portal vein thrombosis	6.9%	22
Biliary leakage	6.2%	20
Intraabdominal haemorrhage	7.5%	24
Biliary stricture	17.8%	57

Ultrasound is the initial imaging technique of choice to assess the early and late complications of LDLT. The examination can be easily performed at the bedside and repeated to follow up detected abnormalities. Most transplant centres, including ours, employ colour Doppler ultrasound in the peri- and post-operative period for assessing the patency of the hepatic artery, hepatic veins and portal veins to ensure proper functioning of the liver graft.

Helical computed tomography (CT) is a valuable complement to ultrasound in the post-operative period and is a safe, accurate and non-invasive means of detecting post-operative complications [28].

### Recipient post-LDLT complications

#### i) Acute rejection

Acute rejection is the most common and most serious complication affecting graft survival. The incidence of acute rejection ranges from 17% to 40% and is lower than that of cadveric liver transplantation with unknown reason [1].

Unfortunately, no imaging modality has proved sensitive or specific for the diagnosis of rejection. The only reliable means of diagnosis is by graft biopsy and histologic study [29].

Imaging studies, including ultrasound, can be used to rule out other complications that have similar clinical signs and symptoms to acute rejection. Ultrasound or CT is occasionally used to guide percutaneous biopsy of the liver graft to confirm the occurrence of acute rejection.

#### ii) Vascular complications

Vascular complications are reported in about 9% of liver transplant patients and are the most common significant post-operative complications. Post-LDLT vascular complications include thrombosis and stenosis of the hepatic artery, hepatic veins and portal veins as well as pseudoaneurysm formation. Vascular problems appear to be more common in the paediatric group of LDLT patients and are most likely due to the small size of vessels and technical factors [1].

Colour Doppler ultrasound is used as the main screening technique, while angiography is reserved for confirming ultrasound findings or when ultrasound findings are equivocal. Doppler spectral waveform analysis provides excellent screening for the detection of hepatic allograft arterial stenosis or thrombosis [30].

The use of ultrasound contrast agents improves visualisation of the hepatic vasculature and shortens the examination time, possibly delaying or obviating the need for more invasive investigations [31].

Excellent results have been reported using multi-slice CT angiography in detecting vascular complications after liver transplantation [32]. Contrast MRA is another alternative non-invasive technique for this purpose [33].

**Figure 2:**
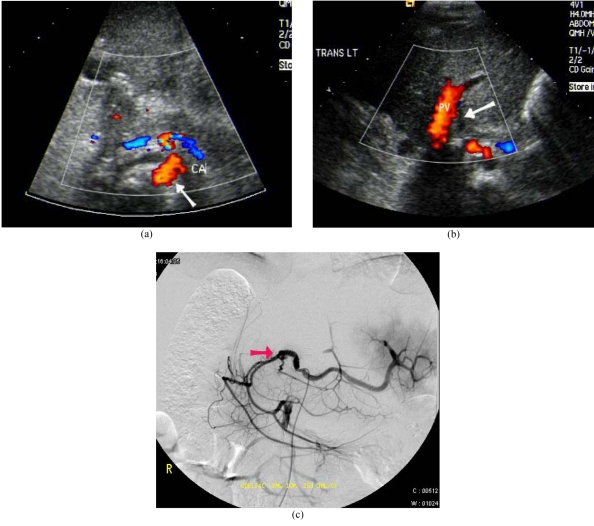
Hepatic artery thrombosis after LDLT. (a) Colour Doppler US showed patent coeliac artery (arrow). (b) Absence of flow and Doppler signal seen at the expected location of the hepatic artery (arrow). PV: Portal vein. (c) Digital subtraction angiogram of the coeliac axis confirmed thrombosis of the hepatic artery (arrow).

Hepatic artery thrombosis is the most common vascular complication of liver transplantation. Treatment requires urgent revascularisation or retransplantation. The diagnosis is established when colour Doppler ultrasound fails to identify both an arterial colour Doppler signal and waveform along the anticipated course of the hepatic artery (Figure 2).

Protocol Doppler ultrasound with RI measurement, during the first two weeks after LDLT, is found to predict hepatic artery thrombosis [34].

The use of contrast enhanced colour Doppler ultrasound may increase the confidence in excluding arterial thrombosis [35].

Definitive diagnosis is by angiography. Contrast-enhanced CT or MRI helps in defining the degree of parenchymal ischaemia in these patients. Geographic areas of decreased echogencity, with preservation of the portal tracts, are the early sonographic signs of hepatic ischaemia [36].

Ultrasound with microbubble contrast enhancement may also provide confirmation of areas of infarction.

**Figure 3:**
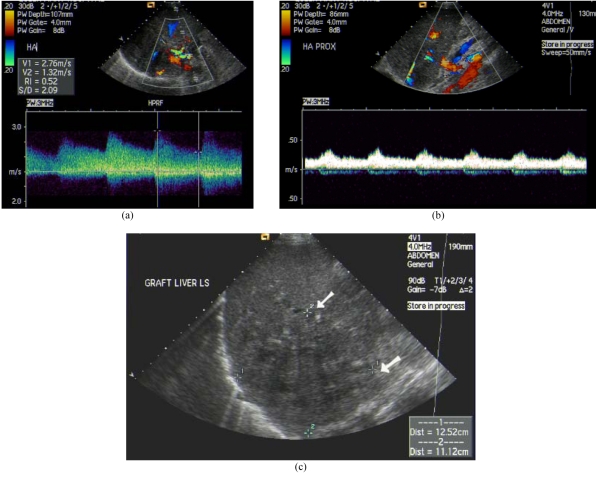
Hepatic artery stenosis after liver transplantation with cholangitic abscess. (a) Colour Doppler US at post-stenotic segment of hepatic artery showed turbulent flow and elevated peak systolic velocity. (b) Tardus-parvus waveform at pre-stenotic segment of hepatic artery with dampened flow. (c) Hypoechoic cholangitic abscess at right lobe liver graft due to hepatic ischaemia (arrows).

The presence of a tardus-parvus waveform pattern in the Doppler ultrasound is suggestive of hepatic artery stenosis (Figure 3) [37].

Contrast-enhanced ultrasound can easily demonstrate focal stenosis at the hepatic arterial anastomosis. Angiography with a view to angioplasty can then be performed. Balloon dilatation of a stenosis is frequently successful, obviating the need for further surgery and vascular reconstruction. Coronary stents may also be used [38].

Hepatic artery pseudoaneurysm is rare after liver transplantation and occurs in 1% of patients. It may be intra- or extra-hepatic in location.

**Figure 4:**
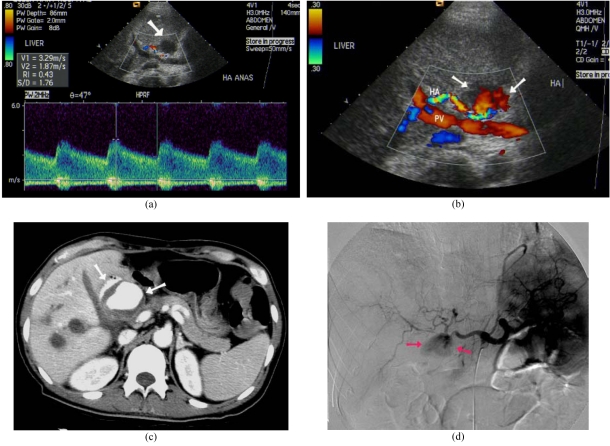
Hepatic artery pseudo-aneurysm after LDLT. (a) Doppler US detected roundish hypoechoic lesion close to hepatic artery (arrow). Spectral Doppler demonstrated elevated peak systolic velocity at neck of pseudo-aneurysm. (b) Colour Doppler US revealed turbulent flow within the lesion (arrows). HA: Hepatic artery, PV: Portal vein. (c) Contrast enhanced CT showed pseudo-aneurysm (arrows). (d) Digital subtraction angiogram of celiac axis demonstrated pseudo-aneurysm (arrows).

Ultrasound is the primary technique for detection, but it may also be seen on contrast-enhanced CT or MRI. Colour Doppler ultrasound shows a high velocity jet with a swirling pattern of flow within the cavity (Figure 4). Treatment is by surgery, coil embolisation or percutaneous thrombin injection [39].

**Figure 5:**
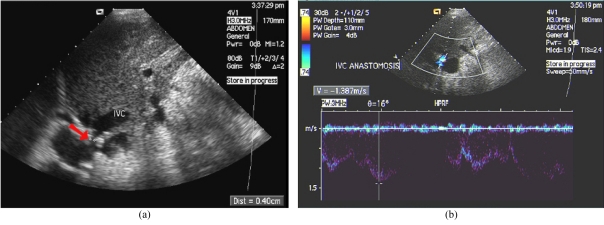
Hepatic vein stenosis after LDLT. (a) US showed stenosis of the hepatic vein (arrow). IVC: Inferior vena cava. (b) Colour Doppler US demonstrated elevated velocity.

A persistent monophasic wave pattern on Doppler ultrasound images of the hepatic veins is suggestive of substantial hepatic vein stenosis after LDLT (Figure 5) [40]. A persistent triphasic wave pattern on Doppler ultrasound images can exclude the possibility of substantial stenosis.

Hepatic vein complications, including thrombosis and stenosis, occur in 1.9% of our patients (Table 1). Hepatic vein thrombosis appears as absence of flow on colour Doppler ultrasound, and echogenic thrombus may be seen within the hepatic vein. Ultrasound with microbubble contrast-enhancement is capable of demonstrating vascular outflow abnormalities of the hepatic veins and IVC when standard colour Doppler examination is difficult or the result is equivocal [31].

**Figure 6:**
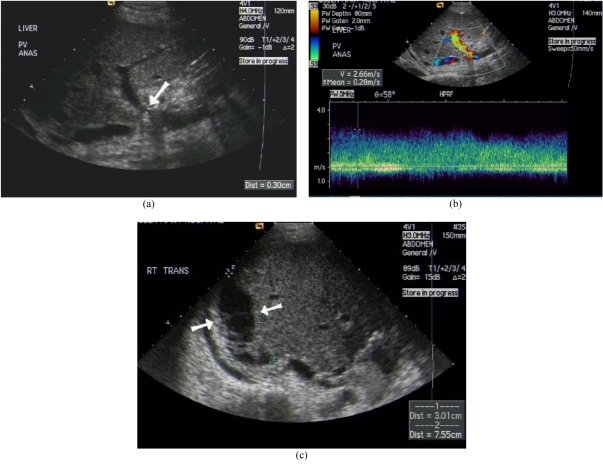
Portal vein stenosis after LDLT with subphrenic collection. (a) US showed stenosis of the portal vein anastomosis (arrow). (b) Colour Doppler US revealed elevated velocity. (c) Subphrenic collection also detected by US (arrows).

Portal vein abnormalities are uncommon and occur in 1% to 3% of transplant recipients. Acute portal vein thrombus appears moderately hypoechoic or isoechoic with absence of Doppler signal [20]. Partial obstruction of the portal vein may be caused by thrombus or stenosis at the portal vein anastomosis (Figure 6). Diffuse low velocities in the portal vein or focally elevated velocities at the anastomosis are signs of portal vein stenosis. Transhepatic portal venoplasty or stent insertion may be performed in significant portal vein stenosis.

#### iii) Biliary complications

**Figure 7:**
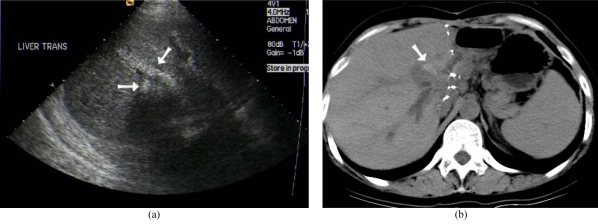
Ductal calculi after LDLT due to biliary stenosis. (a) US showed echogenic ductal calculi at right lobe graft (arrows). (b) Non-contrast CT confirmed hyperdense ductal stone (arrow).

Bile duct complications are a significant cause of post-transplant morbidity and mortality [1], occurring in 24% of our liver transplant recipients (Table 1). Biliary complications are more common in LDLT than in cadveric liver transplantation [41] and include bile leaks, strictures, calculi (Figure 7) or sludge and dysfunction of sphincter of Oddi.

Bililary strictures are more frequent in adult LDLT patients probably due to higher incidence of multiple ducts in right lobe grafts [1]. Percutaneous transhepatic cholangiography or endoscopic retrograde cholangiography is used to diagnose biliary stricture and bile leak. MRC has been found to be a reliable technique for detecting post-orthotopic liver transplanatation biliary complications [42].

Bile leak is caused by technical failure at the duct-to-duct or biliary-enteric anastomosis, and by hepatic artery thrombosis at non-anastomotic sites. Biliary obstruction can be divided into anastomotic and non-anastomotic strictures with causes due to technical failure, ischemia, calculi, mucocele of the cystic duct remnant and recurrent malignancy.

Biliary-enteric anastomosis is performed in many LDLT with the segmental ducts individually anastomosed to a small bowel loop. Anastomotic stenosis can lead to obstruction of a segmental hepatic duct. The ductal dilatation, resulting from biliary stricture, is easily demonstrated by ultrasound. Stricture of the bile duct anastomosis is diagnosed by direct cholangiography, either endoscopic for end to end biliary reconstruction or percutaneous for Roux loop biliary reconstruction.

MRC has a role in non-invasive demonstration of the stricture, reserving the invasive techniques for when intervention is necessary. Treatment is by percutaneous or endoscopic dilatation with temporary stenting, while surgery is reserved for recurrent strictures or those not responding to the less invasive measures.

**Figure 8:**
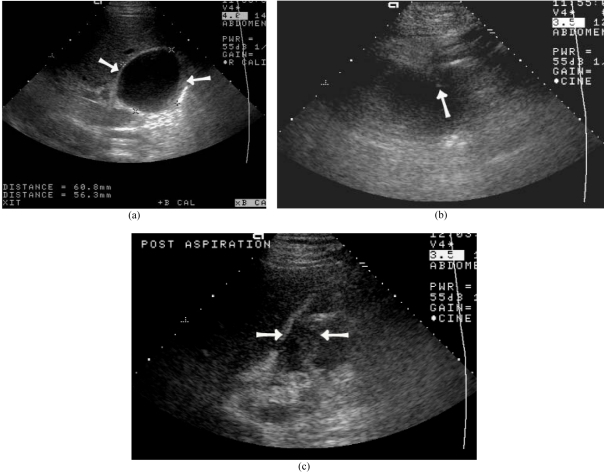
Biloma after LDLT diagnosed by US guided aspiration. (a) Hypoechoic collection detected by US at right lobe (arrows). (b) Tip of needle (arrow) during US guided aspiration (arrow). (c) Post-aspiration US showed marked decrease in size of biloma (arrows).

Bilomas can be aspirated under ultrasound guidance to confirm the diagnosis, promote healing and preserve graft integrity (Figure 8) [43]. Should direct cholangiography confirm a bile leak, then anastomotic stenting may be performed if the integrity of the hepatic artery has been established. Radionuclide (Tc 99m HIDA) and MRC are alternative tools in detecting bile leak. Volumetric mangafodipir-enhanced cholangiography provides satisfactory evaluation of intra-hepatic biliary anatomy. MR evidence of bile leakage is well demonstrated after intravenous infusion of the MR hepatobiliary contrast agent, mangafodipir [44].

#### iv) Post-operative collections

Perihepatic fluid collections and abscesses are not uncommon after LDLT. Intra-hepatic collections appear as cystic or solid masses without internal vascularity and may represent seromas, haematomas or infarction [20].

Complex lesions with mass effect on surrounding structures are suggestive of hematomas. Ultrasound detects these collections and can be used to guide percutaneous drainage in suitable patients.

#### v) Infections

Infections remain the most significant causes of morbidity in patients undergoing LDLT [1], with immuno-suppression being the most critical predisposing factor. Prophylactic regimens vary among transplant centres, but some success can be achieved in reducing the prevalence and severity of peri-operative or long term infections by various viral, fungal, protozoan, bacterial and mycobacterial agents. Serologic examinations and bacterial investigations of blood, sputum, stool, urine and discharge from drains, as well as antibiotic sensitivity tests, should be performed when necessary.

#### vi) Post-transplant malignancies

Approximately 4-5% of liver transplant recipients develop malignant tumours with a four fold increase in the incidence of lymphoma when compared with the general population. The majority of these tumours are non-Hodgkin’s lymphoma. Imaging evaluation and treatment of these diseases follow the principles of accepted oncological staging criteria.

Post-transplant lymphoproliferative disease (PTLD) represents a spectrum of B-cell proliferation ranging from lymphoma to a mononucleosis-like disease. They are found almost exclusively in the paediatric group of LDLT patients [1], and 1.9% of our liver transplant patients are affected (Table 1).

**Figure 9:**
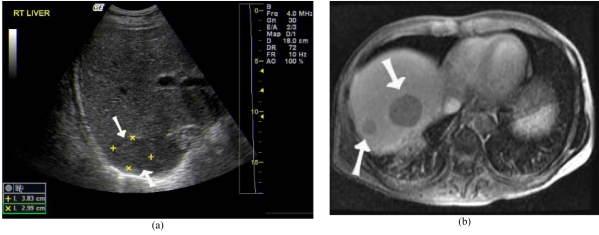
Post transplant lymphoproliferative disease after LDLT. (a) US showed hypoechoic lesion at right lobe graft (arrows). (b) Contrast enhanced T1W MR image revealed hypovascular tumours (arrows).

Imaging features are often non-specific and definitive diagnosis requires biopsy [45]. Ultrasound may reveal hypoechoic, poorly vascularised solid masses in the liver of PTLD patients in addition to the finding of para-aortic lymphadenopathy (Figure 9) [20].

**Figure 10:**
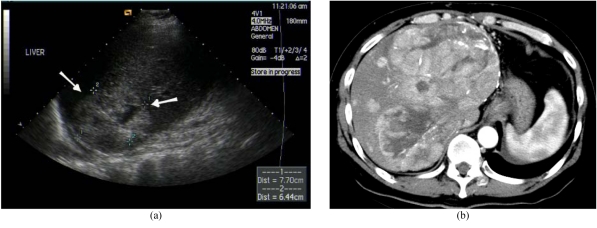
Recurrent hepatocellular carcinoma (HCC) after LDLT. (a) US detected large heterogeneous lesion at liver graft (arrows). (b) Contrast enhanced arterial CT image showed multifocal HCC recurrence.

The use of LDLT in the management of HCC remains controversial. Patients transplanted for HCC are at risk of recurrent disease, with the most frequent sites being the lung and the liver allograft (Figure 10) [46]. Ultrasound can be used to screen these patients and contrast-enhanced ultrasound may increase confidence in difficult cases. Contrast CT or MRI is also effective in the detection of these recurrent tumours.

### Donor post-LDLT complications

The overall donor complication rate for 1,508 LDLT performed in five Asian transplant centres was reported to be 15.8%, and 1.1% of donors underwent re-laparotomy [47].

The complication rate was higher in right lobe than left lobe donors.

More serious complications also occurred in right lobe donors and included cholestasis, bile leakage, biliary stricture, portal vein thrombosis, intra-abdominal bleeding and pulmonary embolism [47].

Radiological work-up follows the accepted imaging criteria. The long term outcome of liver donation remains unknown and transplant centres should continue their follow-up of donors.

## SUMMARY

Living donor liver transplantation (LDLT) provides a valuable alternative to cadaveric liver transplantation in the treatment of acute fulminant liver failure and various end-stage liver diseases.

Ultrasound is the imaging modality of choice in the initial evaluation of both donor and recipient before and after LDLT.

Despite rapid advances in surgical techniques and improvement in immuno-suppression, significant complications still occur after LDLT.

Colour Doppler ultrasound is most useful in the evaluation of post-LDLT vascular complications, while cholangiography is required in the diagnosis of biliary complications.

The knowledge of normal and abnormal ultrasound findings after LDLT is essential for the early detection of post-LDLT complications, permitting timely intervention and ensuring optimal outcome in these patients.

## References

[1] Chen CL, Fan ST, Lee SG (2003). Living-donor liver transplantation: 12 years of experience in Asia. Transplantation.

[2] Tchelepi H, Ralls PW, Radin R (2002). Sonography of diffuse liver disease. J Ultrasound Med.

[3] Llovet JM, Schwartz M, Mazzaferro V (2005). Resection and liver transplantation for hepatocellular carcinoma. Semin Liver Dis.

[4] Hwang S, Lee SG, Joh JW (2005). Liver transplantation for adult patients with hepatocellular carcinoma in Korea: Comparison between cadaveric donor and living donor liver transplantation. Liver Transpl.

[5] Nicolau C, Vilana R, Bru C (2004). The use of contrast-enhanced ultrasound in the management of the cirrhotic patient and for the detection of HCC. Eur Radiol.

[6] Rode A, Bancel B, Douek P (2001). Small nodule detection in cirrhotic livers: Evaluation with US, spiral CT, and MRI and correlation with pathologic examination of explanted liver. J Comput Assist Tomogr.

[7] Low RN, Semelka RC, Worawattanakul S (2000). Extrahepatic abdominal imaging in patients with malignancy: Comparison of MR imaging and helical CT in 164 patients. J Magn Reson Imaging.

[8] Lim HK, Lee WJ, Kim SH (1999). Splanchnic arterial stenosis or occlusion: Diagnosis at Doppler US. Radiology.

[9] Guven K, Acunas B (2004). Multidetector computed tomography angiography of the abdomen. Eur J Radiol.

[10] Schroeder T, Malago M, Debatin JF (2005). “All-in-one” imaging protocols for the evaluation of potential living liver donors: Comparison of magnetic resonance imaging and multidetector computed tomography. Liver Transpl.

[11] Alonso-Torres A, Fernandez-Cuadrado J, Pinilla I (2005). Multidetector CT in the evaluation potential living donors for liver transplantation. Radiographics.

[12] Sahani D, D’Souza R, Kadavigere R (2004). Evaluation of living liver transplant donors: Method for precise anatomic definition by using a dedicated contrast-enhanced MR imaging protocol. Radiographics.

[13] Wang ZJ, Yeh BM, Roberts JP (2005). Living donor candidates for right hepatic lobe transplantation: evaluation at CT cholangiography — initial experience. Radiology.

[14] Kim RD, Sakamoto S, Haider MA (2005). Role of magnetic resonance cholangiography in assessing biliary anatomy in right lobe living donors. Transplantation.

[15] Ayuso JR, Ayuso C, Bombuy E (2004). Preoperative evaluation of biliary anatomy in adult live liver donors with volumetric mangafodipir trisodium enhanced magnetic resonance cholangiography. Liver Transpl.

[16] Lo CM, Fan ST, Liu CL (1999). Minimum graft size for successful living donor liver transplantation. Transplantation.

[17] Fan ST, Lo CM, Liu CL (2000). Safety of donors in live donor transplantation using right lobe grafts. Arch Surg.

[18] Bassignani MJ, Fulcher AS, Szucs RA (2001). Use of imaging for living donor liver transplantation. Radiographics.

[19] Liu CL, Zhao Y, Lo CM (2003). Hepatic venoplasty in right lobe live donor liver transplantation. Liver Transpl.

[20] Chong WK (2004). Ultrasound evaluation of liver transplants. Abdom Imaging.

[21] Marcos A, Fisher RA, Ham JM (1999). Right lobe living donor liver transplantation. Transplantation.

[22] Someda H, Moriyasu F, Fujimoto M (1995). Vascular complications in living related liver transplantation detected with intra-operative and postoperative Doppler US. J Hepatol.

[23] Cheng YF, Huang TL, Chen CL (1998). Intraoperative Doppler ultrasound in liver transplantation. Clin Transplant.

[24] Crossin JD, Muradali D, Wilson SR (2003). US of liver transplants: normal and abnormal. Radiographics.

[25] Garcia-Criado A, Gilabert R, Salmeron JM (2003). Significance of and contributing factors for a high resistive index on Doppler sonography of the hepatic artery immediately after surgery: Prognosis implications for liver transplant recipients. Am J Roentgenol.

[26] Ho MC, Wu YM, Hu RH (2004). Surgical complications and outcome of living related liver transplantation. Transplant Proc.

[27] Saing H, Fan ST, Tam PK (2002). Surgical complications and outcome of pediatric liver transplantation in Hong Kong. J Pediatr Surg.

[28] Quiroga S, Sebastia MC, Margarit C (2001). Complications of orthotopic liver transplantation: spectrum of findings with helical CT. Radiographics.

[29] Boraschi P, Donati F (2004). Complications of orthotopic liver transplantation: Imaging findings. Abdom Imaging.

[30] Dodd GD, Memel DS, Zajko AB (1994). Hepatic artery stenosis and thrombosis in transplant recipients: Doppler diagnosis with resistive index and systolic acceleration time. Radiology.

[31] Berry JD, Sidhu PS (2004). Microbubble contrast-enhanced ultrasound in liver transplantation. Eur Radiol.

[32] Brancatelli G, Katyal S, Federle MP (2002). Three dimensional multi-slice helical computed tomography with the volume rendering technique in the detection of vascular complications after liver transplantation. Transplantation.

[33] Kim BS, Kim TK, Jung DJ (2003). Vascular complications after living related liver transplantation: Evaluation with gadolinium-enhanced three-dimensional MRA. Am J Roentgenol.

[34] Kaneko J, Sugawara Y, Akamatsu N (2004). Prediction of hepatic artery thrombosis by protocol Doppler ultrasonography in pediatric living donor liver transplantation. Abdom Imaging.

[35] Sidhu PS, Marshall MM, Ryan SM (2000). Clinical use of Levovist, an ultrasound contrast agent, in the imaging of liver transplantation: assessment of the pre- and post-transplant patient. Eur Radiol.

[36] Cook GJ, Crofton ME (1997). Hepatic artery thrombosis and infarction: Evolution of the ultrasound appearances in liver transplant recipients. Br J Radiol.

[37] Sidhu PS, Ellis SM, Karani JB (2002). Hepatic artery stenosis following liver transplantation: Significance of the tardus parvus waveform and the role of microbubble contrast media in the detection of a focal stenosis. Clin Radiol.

[38] Denys AL, Qanadli SD, Durand F (2002). Feasibility and effectiveness of using coronary stents in the treatment of hepatic artery stenosis after orthotopic liver transplantation: Preliminary report. Am J Roentgenol.

[39] Patel JV, Weston MJ, Kessel DO (2003). Hepatic artery pseudoaneurysm after liver transplantation: Treatment with percutaneous thrombin injection. Transplantation.

[40] Ko EY, Kim TK, Kim PN (2003). Hepatic vein stenosis after living donor liver transplantation: Evaluation with Doppler US. Radiology.

[41] Maluf DG, Stravitz RT, Cotterell AH (2005). Adult living donor versus deceased donor liver transplantation: A 6-year single centre experience. Am J Transplant.

[42] Boraschi P, Braccini G, Gigoni R (2001). Detection of biliary complications after orthotopic liver transplantation with MR cholangiography. Magn Reson Imaging.

[43] Shaw AS, Ryan SM, Beese RC (2003). Ultrasound of non-vascular complications in the post liver transplant patient. Clin Radiol.

[44] Bridges MD, May GR, Harnois DM (2004). Diagnosing biliary complications of orthotopic liver transplantation with mangafodipir trisodium-enhanced MR cholangiography: comparison with conventional MR cholangiography. Am J Roentgenol.

[45] Scarsbrook AF, Warakaulle DR, Dattani M (2005). Post-transplantation lymphoproliferative disorder: The spectrum of imaging appearance. Clin Radiol.

[46] Ferris JV, Baron RL, Marsh JW (1996). Recurrent hepatocellular carcinoma after liver transplantation: Spectrum of CT findings and recurrence patterns. Radiology.

[47] Lo CM (2003). Complications and long-term outcome of living liver donors: A survey of 1,508 cases in five Asian centres. Transplantation.

